# Wells’ Syndrome Mimicking Angioedema and Revealing a Multiple Myeloma: A Case Report

**DOI:** 10.7759/cureus.82426

**Published:** 2025-04-17

**Authors:** Lamia Mansour Billah, Soumiya Chiheb, Madiha Eljazouly

**Affiliations:** 1 Dermatology and Venerology, Cheikh Khalifa International University Hospital, Mohammed VI University of Health Sciences, Casablanca, MAR; 2 Dermatology Unit, Cheikh Khalifa International University Hospital, Mohammed VI University of Health Sciences, Casablanca, MAR

**Keywords:** dapsone, eosinophilic cellulitis, facial angioedema, multiple myeloma, wells syndrome

## Abstract

Wells’ syndrome (WS), or eosinophilic cellulitis, is a rare inflammatory dermatosis with a variety of clinical presentations. It typically manifests with recurrent pruritic erythematous plaques and edematous swellings, which can be mistaken for other conditions, such as erysipelas or angioedema. We report an unusual presentation of WS localized on the face of a 60-year-old male with a history of recurrent facial plaques, mistakenly treated as angioedema. After an extensive evaluation, a diagnosis of smoldering multiple myeloma was revealed as an underlying neoplastic condition. This case highlights the importance of considering Wells’ syndrome in differential diagnoses of recurrent cellulitis-like presentations, even in the absence of peripheral eosinophilia, and underscores the need for histopathological confirmation for an accurate diagnosis.

## Introduction

This article was previously presented as a meeting abstract at the 2023 EADV (European Academy of Dermatology and Venereology) Congress on October 11, 2023. Wells’ syndrome (WS) is a rare inflammatory dermatosis, often of unknown etiology. Clinical presentations are varied and misleading [1]. Typical features include infiltrated plaques on the extremities, often mistaken for erysipelas or cellulitis. The diagnosis of Wells’ syndrome is based on a set of criteria [2], with histological study playing a key role. Lesions usually regress spontaneously without sequelae. Nevertheless, recurrences remain frequent. Several therapeutic options are available, with corticosteroids being the first-line treatment [3]. Hematological and oncological disorders are often associated with this condition, requiring long-term follow-up [1].

We report a rare localization on the face mimicking angioedema, revealing a smoldering multiple myeloma, which has been successfully treated with dapsone.

## Case presentation

A healthy 60-year-old man presented with an acute onset of pruritic, erythematous plaques, and swelling on the right side of his face and left upper limb. He reported recurrent plaques on the face, which were diagnosed as angioedema over the last four years, resolving short-term systemic corticosteroids and antihistamines.

**Figure 1:**
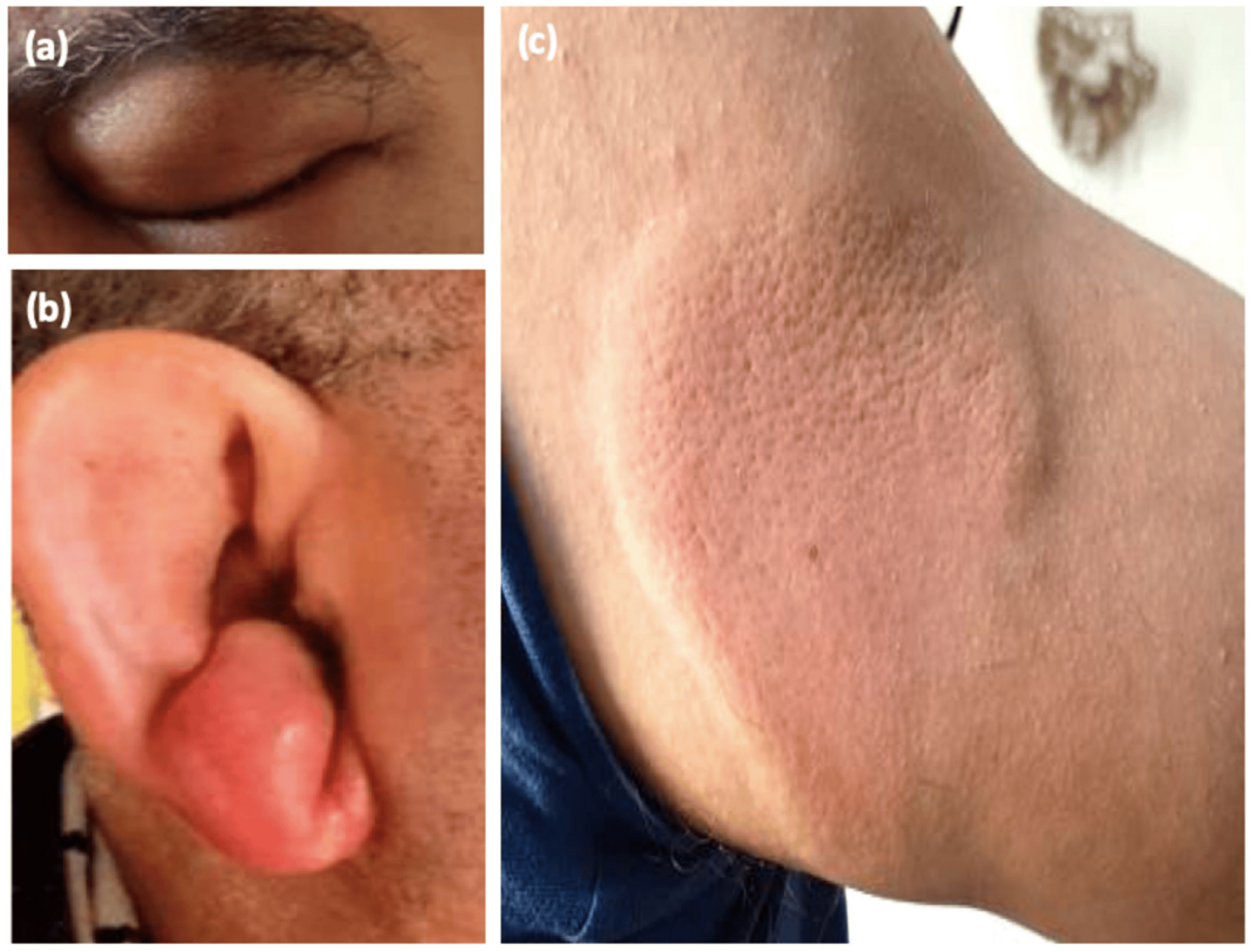
Clinical features (a) Localized, well-demarcated swelling involving the face, eyelids; (b) and the right ear; (c) annular, well-demarcated, red, and infiltrated plaque of the left upper limb

Clinical examination revealed an infiltrated plaque on the face and right ear, as well as an itchy inflammatory annular lesion on the limb (Figure 1). He denied any associated symptoms, drug intake, or insect bites.

**Figure 2:**
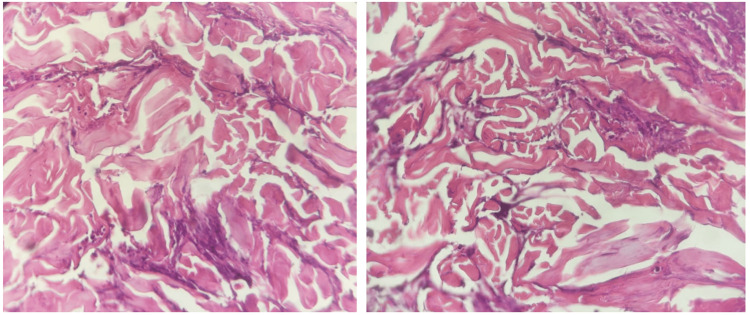
Histopathological features ‘Flame figures’: dermal infiltration by eosinophils (hematoxylin and eosin stain, original magnification ×20)

Biological tests showed an inflammatory syndrome without blood hypereosinophilia. Anatomopathological features described a perivascular and interstitial inflammatory eosinophilic infiltrate essentially with collagen fibers surrounded by eosinophils (flame figure), allowing the diagnosis of WS (Figure 2).

Antinuclear antibodies, anti-DNA, and chest radiography were normal. Laboratory tests for chronic urticaria and angioedema were negative (Table 1). Urinary immunofixation found the presence of albumin and monoclonal light chains of the kappa type. Serum protein electrophoresis revealed an IgG Kappa monoclonal gammopathy, a serum IgG level > 30 g/L, and the presence of dystrophic plasma cells on the myelogram, which correlated with smoldering multiple myeloma (Table 2).

**Table 1:** Basic laboratory investigations ESR: Erythrocyte sedimentation rate, CRP: C-reactive protein, TSH: Thyroid-stimulating hormone, PSA: Prostate-specific antigen, FOBT: Fecal occult blood test (FOBT)

Test description	Observed value	Normal Range
Hemoglobin level (mg/dL)	14.4	13.5 to 17.5
Total leukocyte count (10^3^/μL)	6.9	4.0 to 10.0
Platelet count (10^3^/μL)	166	150 to 450
ESR (mm/h)	8	< 20
Absolute neutrophil count (x10^3^/L)	4.1	2.0 to 7.5
Absolute eosinophil count (x10^3^/L)	0.2	
Reticulocyte count (%)	3.2	> 1.5
Reticulocyte count (%)	3.2	> 1.5
CRP (mg/L)	3	< 5
TSH	1.2	0.4 to 4.0
Antinuclear antibodies	Negative	>1/160
Anti-DNA antibodies	Negative	>1/40
C1 inhibitor level (C1 INH) (mg/dL)	29	21 to 39
C1 inhibitor functional (%)	80	> 70
C1q complement (mg/dL)	14.3	11.8 to 24.4
C4 level (mg/dL)	24	10 to 40
Fecal Calprotectin (µg/g)	12	< 50
Total PSA (ng/mL)	2.8	< 4
FOBT	Negative	-

**Table 2:** Serum immunofixation Ig: Immunoglobulins

Test description	Observed value	Reference interval
Serum total proteins (g/L)	7.3	60-80
Serum Albumin (g/L)	41.2	40.2-47.5
Alpha 1 globulin (g/L)	2.9	2.1-3.5
Alpha 2 globulin (g/L)	5.1	5.0-8.5
Beta 1 globulin (g/L)	3.6	3.4-5.2
Beta 2 globulin (g/L)	2.3	2.0-4.7
Gamma globulin (g/L)	19.1	8.0-13.5
Albumin: Globulin ratio	1.30	1.1 to 2.2
« M » band	Monoclonal band seen (IgG Kappa)	-
Serum IgG levels (g/L)	45	7.0 – 16
Serum IgA levels (g/L)	0.90	0.7 – 4.0
Serum IgM levels (g/L)	0.46	0.4 – 2.3
Free Kappa (light chain) (mg/L)	26.30	3.3-19.4
Free Lambda (light chain) (mg/L)	24.30	5.71-26.3
Free Kappa/Lambda (light chain)	1.08	0.26-1.65

The patient was initially treated with oral corticosteroids at a dose of 1 mg/kg/day, with partial regression of the skin lesions. Due to an incomplete response, dapsone was initiated at 50mg to 100 mg daily, with significant improvement. No recurrence was observed during the two-year follow-up.

## Discussion

Eosinophilic cellulitis, or Wells syndrome, is a rare condition characterized by distinctive clinical and histological features [3]. During the course of the disease, patients present recurrent episodes of acute pruritic dermatitis, persistent urticarial eruptions, and/or painful edematous swellings. Skin lesions may appear as single or multiple, mainly affecting the acral areas [1]. Other clinical patterns have been described, including bullous, papulonodular, vesicular, and fixed drug eruption forms [1,3]. Although the disease typically follows a benign course with spontaneous remissions, recurrences are common and may persist for several years.

Lesions can mimic various dermatoses, including infectious and non-infectious disorders (bacterial cellulitis, annular granuloma, prurigo, urticaria, drug eruptions, erysipelas, insect bites, …). [1-4]. In our case, the unusual feature lies in the clinical presentation of a recurrent infiltrated plaque on the face, mistaken for angioedema, with no triggering factor identified.

The biological assessment should include a complete blood cell count, as blood hypereosinophilia is common [1,4,5]. Nevertheless, it is neither permanent nor required to diagnose the syndrome [3]. Given its large spectrum of clinical features and numerous diagnostic mimickers, the diagnosis is challenging and requires a correlation between clinical and pathological findings.

The histopathology of WS found on biopsy can vary according to disease stage [1,3]. Histopathological findings consistent with WS include dermal edema, eosinophilic major basic protein, and fibrin covering collagen fibers forming the “flame image.” Nevertheless, it is not pathognomonic of WS, as can be found in other diseases (bullous pemphigoid, eczema, parasitic infections, etc.). Our case highlights the need for histopathological analysis in patients with doubtful clinical presentation and no hypereosinophilia to obtain an accurate diagnosis.

The pathogenesis of WS remains unknown [1]. Authors of small case series and individual case reports have suggested a type IV hypersensitivity reaction to a multitude of disorders, including parasitic infections, drug allergies, autoimmune diseases, hematological disorders, and solid tumors [1,6-8]. In our case, a complete workup for systemic involvement revealed a smoldering multiple myeloma. To our knowledge, this is the first described case of smoldering multiple myeloma revealed by Wells syndrome presenting in the misleading form of angioedema.

The management of WS is complex and presents a therapeutic challenge. If an underlying cause exists, causal treatment is required. A case reported in the literature associating Wells syndrome with colorectal cancer showed that hemicolectomy resolved the patient's symptoms [3,9]. Due to its recurrent nature, the first-line treatment consists of systemic corticosteroid therapy [3]. Topical steroids are also an effective alternative to systemic steroids, mainly in children and for localized skin lesions [10,11]. When systemic corticosteroid therapy is not possible, other options such as cyclosporine and dapsone can be considered [12,13].

To date, there are no guidelines specifically designed for WS. Few case reports have featured other anti-inflammatory or immunomodulatory therapies (interferon-α, chloroquine, oral/topical tacrolimus, TNF inhibitors, colchicine, and PUVA therapy) therapy) [1,12,14,15].

## Conclusions

The particularity of our observation lies in its misleading clinical presentation: to our knowledge, WS mimicking angioedema has been described only once in the literature. Physicians need to be aware of this disease in cases of any atypical presentation of recurrent cellulitis not responsive to antibiotics. This case highlights the importance of maintaining a high level of suspicion, even in the absence of peripheral eosinophilia, as it is not sufficient for the diagnosis of the syndrome. A more comprehensive understanding of the pathogenesis, together with larger studies and longer follow-up periods, is required to develop specific treatment guidelines.

## References

[1] Weins AB, Biedermann T, Weiss T, Weiss JM (2016). Wells syndrome. J Deutsche Derma Gesell.

[2] Heelan K, Ryan JF, Shear NH, Egan CA (2013). Wells syndrome (eosinophilic cellulitis): Proposed diagnostic criteria and a literature review of the drug-induced variant. J Dermatol Case Rep.

[3] Räßler F, Lukács J, Elsner P (2016). Treatment of eosinophilic cellulitis (Wells syndrome) - a systematic review. Acad Dermatol Venereol.

[4] Wells GC, Smith NP (1979). Eosinophilic cellulitis. Br J Dermatol.

[5] Moossavi M, Mehregan DR (2003). Wells’ syndrome: a clinical and histopathologic review of seven cases. Int J Dermatology.

[6] Heinig B, Vojvodic A, Lotti T (2019). Wells syndrome - an odyssey. Open Access Maced J Med Sci.

[7] Bansal M, Rai T, Pandey S (2012). Wells syndrome. Indian Dermatol Online J.

[8] Kettani F, Baline K, Hali F (2020). Syndrome de Wells mimant une cellulite bactérienne : un piège diagnostique et thérapeutique. La Revue de Médecine Interne.

[9] Hirsch K, Ludwig RJ, Wolter M (2005). Eosinophilic cellulitis (Wells’ syndrome) associated with colon carcinoma. J Deutsche Derma Gesell.

[10] Reichel M, Isseroff RR, Vogt PJ, Gandour-Edwards R (1991). Wells’ syndrome in children: varicella infection as a precipitating event. Br J Dermatol.

[11] Koh KJ, Warren L, Moore L (2003). Wells’ syndrome following thiomersal‐containing vaccinations. Aust J Dermatology.

[12] Bokotas C, Kouris A, Stefanaki C (2014). Wells syndrome: response to dapsone therapy. Ann Dermatol.

[13] Coelho De Sousa V, Laureano Oliveira A, Cardoso J (2016). Successful treatment of eosinophilic cellulitis with dapsone. DOJ.

[14] Peckruhn M, Tittelbach J, Schliemann S, Elsner P (2015). Life of lesions in eosinophilic cellulitis (Wells’ syndrome)—a condition that may be missed at first sight. Ame Jr Derma.

[15] Long H, Zhang G, Wang L, Lu Q (2016). Eosinophilic skin diseases: a comprehensive review. Clinic Rev Allerg Immunol.

